# A Three, Four or Five Year Course

**Published:** 1922-01

**Authors:** M. D. K. Bremner

**Affiliations:** Chicago


### A THREE, FOUR OR FIVE YEAR COURSE

BY M. D. K. BREMNER, CHICAGO

It is possible that some day in the distant future our people will realize the economic value of national health. They will then be willing to tax themselves for the safeguarding and improvement of the human stock at least as many dollars as they now cheerfully contribute for the protection and development of the animal industry. When that time arrives we will surely have mouth hygiene service in all our public schools where competent oral hygienists will wield the orange-wood stick and draw the tape over the teeth of young America, which will spell the doom of the demon decay and conquer that great enemy of our bicuspids, centrals and molars, misnamed pyorrhea. Then some of us dentists may find it necessary to look for a new avocation. Meanwhile, however, there is a much more serious problem facing us.

How are we going to serve those who need dentistry right now, who are coming into our offices in ever-increasing number? While we have been talking and planning about the education of the public in mouth hygiene and its value as a health measure, the people have gone ahead and picked up a great deal of dental knowledge from various sources, principally magazines and newspapers. Some of the information they have acquired may not be quite correct, but, it is sufficient to make many of them realize the close relationship which exists between mouth conditions and systematic health. The physicians also awoke to the fact that the oral cavity is a part of the human anatomy, indeed, some medical men seem to have forgotten that there are any other organs in the body except teeth and gums.

Incidentally, the dental profession has also made some progress. We, too, have learned a few things. For example, we know now that mummifying paste is not a good root canal filling; that "gum-boils" are not altogether harmless; that a mass of plaster in a shovel size tray does not make an ideal impression for an artificial denture. The methods of practice have changed very materially and the change has been in the direction of greater thoroughness and more exactness, which means, of course, the consumption of more time.

With the advent of the X-ray and research work which has been done during recent years in almost every branch of dentistry, the conscientious operator can no longer work in the haphazard fashion of the past. It takes time to prepare a cavity scientifically, to maintain asepsis while filling root canals, or to scale a set of teeth properly. The result, therefore, is that while the demand for dental services has increased, our ability to satisfy this demand has decreased.

The remedy which suggests itself is an addition to our ranks. This can be done only by encouraging an enrollment of young men and young women into the dental colleges; instead of that, however, many schools are insisting upon lengthening the course, making it five years; thereby reducing the number of dentists, and still further diminishing the dental services available for the people.

Without entering into a discussion whether or not the fifth year is necessary altogether; whether a man must really spend five years in a school before he may be pronounced fit to enter upon the practice of dentistry, what I would like to know is this: Is it safe at this time to reduce the number of dentists?—*Dental Facts.*

